# Phylogenetic Analysis of Local-Scale Tree Soil Associations in a Lowland Moist Tropical Forest

**DOI:** 10.1371/journal.pone.0013685

**Published:** 2010-10-27

**Authors:** Laura A. Schreeg, W. John Kress, David L. Erickson, Nathan G. Swenson

**Affiliations:** 1 Department of Biology, School of Natural Resources and Environment, University of Florida, Gainesville, Florida, United States of America; 2 Smithsonian Tropical Research Institute, Balboa, Ancon, Republic of Panama; 3 Department of Botany, National Museum of Natural History, Smithsonian Institution, Washington, D. C., United States of America; 4 Department of Plant Biology, Michigan State University, East Lansing, Michigan, United States of America; Centre National de la Recherche Scientifique, France

## Abstract

**Background:**

Local plant-soil associations are commonly studied at the species-level, while associations at the level of nodes within a phylogeny have been less well explored. Understanding associations within a phylogenetic context, however, can improve our ability to make predictions across systems and can advance our understanding of the role of evolutionary history in structuring communities.

**Methodology/Principal Findings:**

Here we quantified evolutionary signal in plant-soil associations using a DNA sequence-based community phylogeny and several soil variables (e.g., extractable phosphorus, aluminum and manganese, pH, and slope as a proxy for soil water). We used published plant distributional data from the 50-ha plot on Barro Colorado Island (BCI), Republic of Panamá. Our results suggest some groups of closely related species do share similar soil associations. Most notably, the node shared by Myrtaceae and Vochysiaceae was associated with high levels of aluminum, a potentially toxic element. The node shared by Apocynaceae was associated with high extractable phosphorus, a nutrient that could be limiting on a taxon specific level. The node shared by the large group of Laurales and Magnoliales was associated with both low extractable phosphorus and with steeper slope. Despite significant node-specific associations, this study detected little to no phylogeny-wide signal. We consider the majority of the ‘traits’ (i.e., soil variables) evaluated to fall within the category of ecological traits. We suggest that, given this category of traits, phylogeny-wide signal might not be expected while node-specific signals can still indicate phylogenetic structure with respect to the variable of interest.

**Conclusions:**

Within the BCI forest dynamics plot, distributions of some plant taxa are associated with local-scale differences in soil variables when evaluated at individual nodes within the phylogenetic tree, but they are not detectable by phylogeny-wide signal. Trends highlighted in this analysis suggest how plant-soil associations may drive plant distributions and diversity at the local-scale.

## Introduction

An important goal in tropical ecology is to identify the strength of plant associations with their physical environments in order to understand the factors underlying the distribution and abundance of species. Identifying plant-soil associations is not a new goal (for example, see studies reviewed in [1]). Many advances have been made at the levels of landscape and mesoscale (10^2^–10^4^ km^2^ and 1–100 km^2^ respectively, as defined by [2], e.g. [3], [4], [5], [6], [7], [8], [9]), but progress in understanding local-scale (<1 km^2^) associations in the tropics has been slower for two reasons. First, intensive inventories are required to quantify the spatial distributions and abundances of tropical tree communities. Second, quantitative measurements of the abiotic environment are required at a high spatial resolution within the tree communities being inventoried. Work in Malaysia has succeeded in confronting both of these obstacles by mapping fine scale variation in soil texture [10], [11], which can correlate with soil resources, within a 52-ha plot. Similarly, recent work on Barro Colorado Island (BCI) in the Republic of Panamá has mapped the spatial distribution of soil nutrients and elements within the 50-hectare forest dynamics plot on the island [2]. The results of this work have shown that indeed some tree species in the BCI plot had significant associations with soil nutrients and elements.

Here we expand on previous work on local-scale plant-soil associations by moving the analyses beyond the species-level associations and investigating the degree to which closely related species share similar associations for soil variables on a local-scale. Such an approach may be useful for understanding local-scale patterns that exist across highly diverse tropical forests that may share few species but have ‘deeper’ phylogenetic overlap (e.g., shared genera and families). For example, physiological traits which may influence plant distributions are known to exist at phylogenetic nodes for aluminum accumulation (e.g., Vochysiaceae and Melastomataceae). This trait can serve as a mechanism of aluminum tolerance [12] and it is reasonable to hypothesize that, in some systems, it could translate into local-scale plant-soil associations for those groups. Similarly, node-specific plant associations could exist for other elements, including nutrients that may be limiting for plant productivity. In highly weathered soils in Hawaii [13] phosphorus has been shown to be a limiting nutrient and could be limiting to different degrees, perhaps on a taxon-specific basis, in other tropical forests.

The degree to which soil nutrient and element associations are similar amongst closely related species is also of practical importance for phylogenetic investigations into the community structure [14], [15], [16], [17], [18], [19], [20]. Recent work has shown that within some topographically defined habitats of the BCI forest plot, species assemblages tend to be more similar in their phylogenetic composition than expected [16], [19]. Knowledge regarding the degree to which some closely related species occur on similar positions on soil resource gradients may help uncover mechanisms underlying empirical patterns of community phylogenetic structure.

Although it is not possible to conclusively determine that associations are the result of soil influencing plant distribution, as opposed to plants influencing soil variables, there are a few reasons why the sampling design of the BCI soil nutrient and element data set is more likely to detect plant responses to soil variation. As outlined by others [2], the high tree diversity homogenizes inputs, decreasing feedbacks of litter quality on soil properties. In addition, soil nutrients and elements are correlated with topography suggesting geological processes underlie nutrient and element variation in the plot. Furthermore, soil sampling was intensive but, due to the size of the plot area, data analysis is dependent on kriging between the 50 m×50 m (plus 3 random samples at alternate grid points) sampling locations to create estimates at a scale of 20 m×20 m. Considering this spatial scale, along with the high number of small individuals in the data set (∼90 percent of the individuals in the BCI plot are less than 10 cm in diameter), strengthens the argument that associations are more likely to be due to soil influences on plant distribution than the reciprocal effect. However, an important caveat to this reasoning is when species are highly aggregated, in which case many individuals could have a cumulative effect on soil properties. Within the BCI 50-ha plot, it has been shown that most species are spatially aggregated but there is large variation in the degree of aggregation [21].

The present study used a phylogenetic hypothesis of the relationships among the taxa in the BCI forest plot to quantify the evolutionary signal in three soil nutrients and elements (extractable phosphorus, aluminum and manganese), pH, slope and a general index of soil fertility determined by using Principal Component Analyses (PCA) axes which combine 15 soil variables. Our main hypothesis was: if the evolution of soil associations has been relatively conserved for a given soil variable or group of variables (as evaluated through PCA axes), there will be node-specific signal in soil variables, suggesting that the association between edaphic variation and local-scale plant distribution within the BCI plot exist beyond the species-level. The null hypotheses being: an absence of signal in the soil variables. This would suggest that local-scale variation of edaphic factors are not important in determining distributions of clades of species; however, the associations between plant distributions and soil factors can still exist for individual species (as shown in [2]).

In contrast to our expectations for node-specific signal, we did not expect to find phylogeny-wide signal for extractable aluminum, manganese, phosphorus, or slope (to the extent that it is a proxy for water), due to the nature of these metrics. We are interested in whether groups of species show association with soil variables, which serve as the ‘trait’ values for the phylogenetic analyses. These are not trait values in the traditional sense, as they are not directly measured on individual organisms. However, the soil variables have the potential to represent the evolutionary history of an organism and we consider them to be ecological ‘traits’, which is one category of traits highlighted in Blomberg et al. [22], along with morphological, life history, physiological, and behavioral. For example, Blomberg et al. [22] consider mean annual temperature and seasonality as ecological traits which, similar to soil variables, are abiotic factors that could influence an organisms range of potential environments and competitive ability.

Here we are investigating if taxa show tolerance, or intolerance, within the ranges of soil variables. It follows that, within a group, species exhibiting tolerance or intolerance would have average values of the soil metric of interest clustered toward the extreme of the gradient compared to those expected by chance. For tolerance, the shifted average would reflect either 1) the ability to occupy the more extreme sites while also existing on less extreme sites or 2) the limited distribution on the tail of the tolerance gradient due to a trade-off decreasing their presence in the rest of the gradient. In contrast, data for less tolerant taxa would extend over a range of less extreme values. Therefore, phylogeny-wide signal based on soil variables would be unlikely to be significant because well constrained trait values throughout the phylogeny, which might be expected for morphological or physiological traits, are not expected for this ecological trait. However, the phylogeny could show node-specific associations in the trait value (as hypothesized above).

## Methods

Barro Colorado Island (BCI) is a semideciduous moist forest with average rainfall of 2,600 mm [23]. In the 50-ha plot, red light clays are the most abundant soil category but brown fine loams are also present [24] (for more information on BCI soils see [25]). Census data for the 50-ha plot was collected by Smithsonian Tropical Research Institute (STRI) Forest Dynamics project, lead by R. Condit, S. Hubbell and R. Foster [26], [27]; data can be requested at http://ctfs.si.edu/datasets/bci/) and we used data from the 2005 census. All trees ≥1cm diameter are included in the census and the sampling protocol is described in [28].

For soil traits, we focus on previously published data on extractable phosphorus, aluminum, manganese, pH, and slope. We also use a general index of soil fertility using PCA axes created from analysis of 15 soil variables. Soil collection and analysis was conducted by J. Dalling, K. Harms, R. John, R. Stallard, and J. Yavitt [2], [29]. Methods are described in [30]. Briefly, 300 samples were collected to 10 cm depth following a 50 m×50 m grid (plus 3 random samples at alternate grid points) and soil analyses included extractable aluminum, boron, calcium, copper, iron, potassium, magnesium, manganese, inorganic nitrogen, phosphorus and zinc, in addition to net nitrogen mineralization rates, and pH. These data were then used to create estimates at a scale of 20 m×20 m. Nitrogen was extracted using potassium chloride and estimated colorimetrically using an autoanalyzer, while all other nutrients and elements were extracted with a Mehlich-3 solution [31] and analyzed using inductively coupled plasma-atomic emission spectrometry (ICP-AES) at Cornell University Laboratories [2]. Net nitrogen mineralization rates represent nitrogen mineralized during a 28 day in situ incubation. Soil pH was measured using a 1∶3 ratio of field moist soil to distilled water [30]. In addition, although slope and elevation are topographic and not soil characteristics, we included them as soil variables to guide inference about the importance of soil water. For example, on BCI steep slopes have been shown to have higher water potentials than plateau areas [32], [33]. The slope and elevation for each 400 m^2^ subplot inside the BCI forest plot were downloaded with the original 2005 census data.

### Supertree and trait files

The present study uses a previously published molecular phylogeny for the BCI forest plot published by [19]. The phylogeny was generated using a three-locus (*rbcL*, *trnH-psbA*, and *matK*) DNA barcode dataset generated for the plot. The sequencing, alignment and phylogenetic inference methods are discussed in detail elsewhere [19]. The phylogeny resolves the majority of the nodes with the phylogeny and provides a marked improvement over phylogenetic supertrees that paste species onto phylogenetic backbones using the taxonomic hierarchy [34].

To generate the species by soil variable association data set we first made geographic information system (GIS) map layers of each of the soil variables. Next, we performed a principal component analysis (PCA) to investigate suites of correlated soil variables. Data input into the PCA analysis were log transformed when necessary (i.e. extractable B, Ca, Cu, Fe, K, Mg, P and Zn, and elevation) and scaled. The resulting PCA scores were used to generate GIS map layers for the first three PCA axes. Next using the x-y coordinates in the original BCI census file we plotted the BCI tree data in the GIS on top of the soil variable maps and extracted the soil variable value for each individual stem. Then for each species we calculated the median value on which each species was found for each soil variable. Phylogenies and trait files for medians were created for all individuals ≥1 cm in diameter.

### Phylogenetic signal

Phylogenetic signal is defined as the tendency for related species to resemble each other more than they resemble species drawn at random from the tree [22] and phylogenetic signal is often calculated using the mean contrast value for all nodes descendant of a node [22], [35], [36], [37]. In this analysis we use three metrics (phylogeny-wide signal, node-specific signal and Blomberg's K statistic) to measure the tendency of related species to resemble each other more than they resemble species drawn at random. Phylogeny-wide signal indicates if a pattern was detected across the entire phylogeny, while node-specific signal indicates if significant associations were detected for nodes within the phylogeny. Bloomberg's K statistic was used to quantify phylogenetic signal in the soil data and to compare the magnitude of effect sizes among traits as measured from the root node [38]. For consistency, we only refer to the metrics which use contrast values as phylogenetic signal (i.e. the phylogeny-wide signal and Bloomberg's K statistic; see below). We use the term evolutionary signal to refer to all three metrics, including node-specific signal.

The analysis of traits (AOT) module in the software Phylocom v4.0 [39] was used to test for phylogeny-wide signal and node-specific signal in soil variables. In the AOT procedure, phylogeny-wide signal was evaluated at the root node, while node-specific signal was tested for each node in the phylogeny. Phylogeny-wide signal here is based on the mean contrast value for all nodes descendant of a node, while node-specific signal is based on means of all taxa subtended by the node of interest. The analysis for Blomberg's K statistic was performed using the R package Picante [40].

To be conservative with interpreting significant node-specific results from AOT module, in this paper we focus on nodes that were significant for both tip and ancestral averaging. Detailed information and references for all AOT can be found in [37]. The observed mean value of each node is compared to a null distribution of mean values that is generated by permutating trait values across the tips of the phylogeny 999 times. If the observed value for a node lands in the p = 0.05 tails of the null distribution, the trait has significant signal at that node and all descendant taxa have average trait values more similar than expected.

High and low tail tests were conducted for node-specific associations of all traits. However, for PCA axes, only associations with the high tail tests are considered. Significant associations with low values of a PCA axis are not biologically meaningful and therefore are not included in the results. Considering the number of species and the number of individuals per species for a given node is important for identifying significant node-trait associations that may be skewed by poor representation of one or several taxa. To address this, we list the number of individuals per species for each node of interest. Significant nodes that are dependent on a species that has less than five individuals are not included in the results and nodes representing large numbers of individuals are given emphasis during interpretation of the results.

## Results

### Principal components analysis of soil traits

The first three PCA axes were found to represent 74% of the total variance among the 15 soil variables used in this analysis, with the first axis representing more than three times more variance than the second (Table 1). The fourth axis explained only 8%, while the fifth explained only 3% of the variance. Therefore, only the first three axes are used as phylogenetic analysis ‘traits’ to represent combinations of soil variables that were well represented within the plot. Our analysis used additional variables compared to the PCA results reported in [2] (Table 1), leading to different results, most notably of axes 2 and 3.

**Table 1:** Loadings for the first three axes of principal components analysis (PCA) and the mean and range of the soil variables in 20 m×20 m data set.

Soil variable	Axis 1	Axis 2	Axis 3	Trait mean and range
Aluminum	0.142	0.271	0.45	1012.1 (283.2–1563.8)
Boron	−0.3	0.169	0.169	0.93 (0.04–3.33)
Calcium	−0.310	0.193	0.271	1697.3 (343.5–4416.5)
Copper	−0.234	0.255	0.155	7.1 (1.3–15.1)
Iron	−0.219	0.207	−0.226	178.0 (51.1–350.0)
Potassium	−0.367	0.143	−0.469	167.4 (58.2–391.1)
Magnesium	−0.274	0.211	0.389	296.3 (43.9–861.3)
Manganese	−0.285	0.522	0.436	368.2 (9.4–781.5)
Phosphorus	0.782	−0.113	−0.248	2.87 (0.30–8.30)
Zinc	−0.355	−0.132	0.115	5.1 (0.9–17.9)
Nitrogen	−0.103	0.291	0.257	25.9 (12.5–47.1)
Nitrogen mineralization	−0.459	−0.692	−0.224	17.9 (−11.7–47.6)
pH	−0.144	−0.109	−0.116	4.67 (3.64–5.35)
Elevation	0.122	−0.188	0.211	144.4 (121.3–159.3) m
Slope	−0.202	−0.409	0.211	4.33 (0.02–16.66) degrees
% variance	51%	13%	10%	

Soil variables include Mehlich-3 extractable elements, potassium chloride extractable inorganic nitrogen, nitrogen mineralization rates, pH, slope and elevation. All elements are expressed as mg element kg^−1^ dry soil. Nitrogen mineralization rates are expressed as mg N mineralized kg^−1^ soil per 28 days. Percent variance for each axis is included.

### Signal in plant-soil associations

Of the eight soil variables evaluated (medians of four soil variables, slope, plus 3 PCA axes), none were found to have a significant phylogeny-wide phylogenetic signal (p<0.05; data not shown). Only two traits (PCA axis 2 and 3, Table 2) were found to have a K statistic greater than unity. The lack of significance for most variables suggests that, at the phylogeny-wide level, closely related species do not have more similarity than expected by Brownian motion model of trait evolution. Thus, across the entire phylogeny there was little to no phylogenetic signal in species soil associations in the BCI forest plot.

**Table 2:** Bloomberg's K statistic for soil traits.

Trait	K	*P* value
Al	0.80	0.091
Mn	0.77	0.102
P	0.92	**0.010**
pH	0.64	0.694
elevation	0.88	0.091
slope	0.83	0.146
PCA axis 1	0.69	0.556
PCA axis 2	1.45	**0.003**
PCA axis 3	1.06	**0.044**

Aluminum, manganese and phosphorus were extracted with Mehlich-3. See Table 1 for information on PCA axes. Significant results of a two tailed test are shown in bold.

Although the analysis lacked phylogenetic signal across the entire phylogeny, a number of significant signals at individual nodes (i.e., node-specific signals) were detected. Rate of significance was 3.8%, which is less than that expected given the number of tests conducted. Despite this, we do report on those instances where groups of closely related species share similar soil associations. Most notably, the node shared by the Laurales and Magnoliales was significantly associated with low extractable phosphorus (Table 3, Figure S1), high slope (Table 4) and PCA axis 3 (Table 5). *Protium* (in Burseraceae) was also associated with low extractable phosphorus (Table 3). In contrast, Apocynaceae and nodes uniting groups within Apocynaceae were found to be associated with higher levels of extractable phosphorus (Table 4).

**Table 3:** Significant node-specific evolutionary signals for low tail tests of trait (i.e. soil variable) medians.

Trait	Node shared by:	Family	Individuals per species	T mean	T p-value
P	Protium*	Burseraceae	2829, 698, 2853, 9	1.87	0.05
P	Laurales & Magnoliales*		367, 27, 179, 112, 131, 2, 237, 162, 70, 59, 2115, 183, 1394, 44, 621, 472, 11327, 896, 123, 485, 794	2.19	0.02
pH	Salicaceae & Clusiaceae		6, 24, 124, 34, 410, 26, 131, 14, 434, 67, 484, 23, 4, 41, 152, 393, 4602, 391, 1427	4.39	0.03
pH	Salicaceae*		6, 24, 124, 34, 410, 26, 131, 14, 434	4.02	0.002
pH	*Casearia commersoniana C. arborea*, *Zuelania guidonia*, *Laetia thamnia*, *L. procera*, *C. sylvestris*, *C. guianensis*, *C. aculeata*	Salicaceae	24, 124, 34, 410, 26, 131, 14, 434	3.99	0.004
pH	*Casearia arborea*, *Zuelania guidonia*, *Laetia thamnia*, *L. procera*, *C. sylvestris*, *C. guianensis*, *C. aculeata*	Salicaceae	124, 34, 410, 26, 131, 14, 434	3.94	0.006
pH	*Zuelania guidonia*, *Laetia thamnia*, *L. procera*, *Casearia sylvestris*, *C. guianensis*, *C. aculeata*	Salicaceae	34, 410, 26, 131, 14, 434	3.84	0.008
pH	*Zuelania guidonia*, *Laetia thamnia*	Salicaceae	34, 410	2.24	0.006
pH	*Psychotria marginata* & P. graciliflora*	Rubiaceae	581, 53	4.37	0.044
slope	*Ficus obtusifolia* & *F. costaricana*	Moraceae	6, 8	2.13	0.046
slope	Salicaceae*		See above	2.92	0.026
slope	*Amaioua corymbosa*, *Borojoa panamensis*, *Alibertia edulis*	Rubiaceae	22, 1, 370	2.17	0.008
slope	*Psychotria horizontalis*, *P. marginata*, *P. graciliflora*	Rubiaceae	3119, 581, 53	2.38	0.024
slope	*Thevetia ahouai*, *Tabernaemontana arborea*, *Stemmadenia grandiflora*, *Rauvolfia littoralis*, Lacmellea panamensis*	Apocynaceae	91, 1593, 1, 1, 100	2.40	0.008
slope	*Tabernaemontana arborea*, *Stemmadenia grandiflora*, *Rauvolfia littoralis*, Lacmellea panamensis*	Apocynaceae	1593, 1, 1, 100	2.54	0.024
slope	*Elaeis oleifera*, *Chamaedorea tepejilote*, Attalea butyracea*	Arecaceae	21, 7, 34	2.28	0.024

All nodes listed were significant for both ancestral weighted trait and tip averaging analyses using data for all stems ≥1cm. Tip (T) averaging means for Mehlich-3 extractable soil elements are shown in units of mg kg^−1^ soil and slope is in degrees. Significant nodes which are dependent on a species that has less than five individuals are not shown. Nodes which were significant for more than one of the variables investigated are indicated with an asterisk. See footnotes of Table 5 for a summary of nodes with multiple associations.

**Table 4:** Significant node-specific evolutionary signals for high tail tests of trait medians.

Trait	Node shared by:	Family	Individuals per species	T mean	T p-value
Al	*Ficus trigonata*, *F. popenoei*, *F. obtusifolia*, *F. costaricana*, *F. citrifolia*, *F. bullenei*	Moraceae	5, 3, 6, 8, 1, 5	1172.53	0.002
Al	*Ficus popenoei*, *F. obtusifolia*, *F. costaricana*, *F. citrifolia*, F. bullenei*	Moraceae	3, 6, 8, 1, 5	1192.98	0.002
Al	Lythraceae, Vochysiaceae, Myrtaceae		5, 27, 60, 46, 1816, 482, 1751, 611, 449	1130.94	0.002
Al	Vochysiaceae & Myrtaceae		27, 60, 46, 1816, 482, 1751, 611, 449	1110.38	0.024
Al	*Eugenia & Chamguava*	Myrtaceae	1816, 482, 1751, 611, 449	1116.23	0.044
Al	Psychotria marginata & P. graciliflora*	Rubiaceae	See Table 3	1196.71	0.034
Mn	*Poulsenia armata*, *Olmedia aspera*, Maquira costaricana*	Moraceae	1162, 149, 1396	454.64	0.022
Mn	*Zanthoxylum setulosum*, *Z. panamense*, *Z. ekmanii*	Rutaceae	1, 178, 194	449.53	0.028
Mn	Protium*	Bursuraceae	See Table 3	433.05	0.046
Mn	*Psychotria limonensis*, *P. granadensis*, *P. psychotriifolia*, *P. chagrensis*	Rubiaceae	65, 3, 1, 13	445.75	0.014
P	*Ficus popenoei*, *F. obtusifolia*, *F. costaricana*, *F. citrifolia*, F. bullenei*	Moraceae	See above	3.52	0.012
P	*Miconia impetiolaris*, *M. elata*, *M. dorsiloba*, *Clidemia septuplinervia*	Melastomataceae	14, 12, 2, 3	3.45	0.048
P	Apocynaceae		90, 1593, 1, 1, 100, 469	3.73	0.008
P	*Thevetia ahouai*, *Tabernaemontana arborea*, *Stemmadenia grandiflora*, *Rauvolfia littoralis*, Lacmellea panamensis*	Apocynaceae	See Table 3	3.90	0.006
P	*Tabernaemontana arborea*, *Stemmadenia grandiflora*, *Rauvolfia littoralis*, Lacmellea panamensis*	Apocynaceae	See Table 3	3.78	0.02
P	*Bactris major*, *B. barronis*, *Elaeis oleifera*, *Chamaedorea tepejilote*, *Attalea butyracea*	Arecaceae	80, 5, 21, 7, 34	3.73	0.004
P	*Elaeis oleifera*, *Chamaedorea tepejilote*, Attalea butyracea*	Arecaceae	See Table 3	3.86	0.024
pH	*Sapium aucuparium*, *S. ‘spnov’*, *Hura crepitans*	Euphorbiaceae	52, 3, 103	4.89	0.02
pH	Meliaceae		11344, 478, 1774, 59, 823, 10	4.82	0.046
slope	*Poulsenia armata*, *Olmedia aspera*, Maquira costaricana*	Moraceae	See above	6.07	0.04
slope	Laurales & Magnoliales*		See Table 3	4.63	0.032
slope	Piper	Piperaceae	17, 5, 131, 16, 50, 19, 27	5.47	0.012
slope	*Piper perlasense*, *P. colonense*, *P. reticulatum*, *P. imperialis*, *P. cordulatum*, *P. aequale*	Piperaceae	17, 5, 131, 16, 50, 19	5.45	0.02

See Table 3 for column details.

**Table 5:** Significant node-specific evolutionary signals for high tail tests of PCA axes.

Trait	Node shared by:	Family	Individuals per species	T mean	T p-value
PCA axis 1	Solanaceae, Bignoniaceae, Acanthaceae, Verbenaceae		69, 5, 4, 7, 230, 71, 5, 8, 280, 44	0.26	0.012
PCA axis 1	Bignoniaceae, Acanthaceae, Verbenaceae		230, 71, 5, 8, 280, 44	0.27	0.034
PCA axis 1	Psychotria marginata & P. graciliflora*	Rubiaceae	See Table 3	0.38	0.036
PCA axis 2	*Ficus popenoei*, *F. obtusifolia*, *F. costaricana*, *F. citrifolia*, F. bullenei*	Moraceae	See Table 4	0.11	0.004
PCA axis 2	*Thevetia ahouai*, *Tabernaemontana arborea*, *Stemmadenia grandiflora*, *Rauvolfia littoralis*, Lacmellea panamensis*	Apocynaceae	See Table 3	0.14	0.006
PCA axis 2	*Tabernaemontana arborea*, *Stemmadenia grandiflora*, *Rauvolfia littoralis*, Lacmellea panamensis*	Apocynaceae	See Table 3	0.15	0.006
PCA axis 3	*Pseudobombax septenatum & Ochroma pyramidale*	Malvaceae	35, 10	0.13	0.038
PCA axis 3	Laurales & Magnoliales*		See Table 3	0.04	0.002
PCA axis 3	Laurales		367, 27, 179, 112, 131, 2, 237, 162, 70, 59, 2115	0.05	0.026
PCA axis 3	*Siparuna*	Siparunaceae	376, 27	0.13	0.032
PCA axis 3	*Piper colonense*, *P. reticulatum*, *P. imperialis*, *P. cordulatum*, *P. aequale*	Piperaceae	5, 131, 16, 50, 19	0.11	0.002
PCA axis 3	*Piper reticulatum*, *P. imperialis*, *P. cordulatum*, *P. aequale*	Piperaceae	131, 16, 50, 19	0.10	0.02

See Table 3 for column details.

Summary of nodes with multiple associations:

*Ficus popenoei*, *F. obtusifolia*, *F. costaricana*, *F. citrifolia*, *F. bullenei*: high Al, P, and PCA axis 2.

*Poulsenia armata*, *Olmedia aspera*, *Maquira costaricana*: high Mn and slope.

Salicaceae: low pH and slope.

*Protium*: low P, high Mn.

*Psychotria marginata* & *P. graciliflora*: low pH, high Al and PCA axis 1.

*Thevetia ahouai*, *Tabernaemontana arborea*, *Stemmadenia grandiflora*, *Rauvolfia littoralis*, *Lacmellea panamensis*: low slope, high P and PCA axis 2.

*Tabernaemontana arborea*, *Stemmadenia grandiflora*, *Rauvolfia littoralis*, *Lacmellea panamensis*: low slope, high P and PCA axis 2.

Laurales & Magnoliales: low P, high slope and PCA axis 3.

*Elaeis oleifera*, *Chamaedorea tepejilote*, *Attalea butyracea*: low slope, high P.

Node-specific results for association with extractable aluminum and manganese, both potentially toxic elements, were intriguing. The most recent common ancestor node for Myrtaceae and Vochysiaceae, in addition to the node shared by *Eugenia* and *Chamguava* (in Myrtaceae) and a number of nodes shared by species of *Ficus* (in Moraceae) showed association with high extractable aluminum (Table 4, Figure S2). While the *Ficus* results are based on a small number of individuals, the node shared by Myrtaceae and Vochysiaceae and the node hosting *Eugenia* and *Chamguava* are based on robust sample sizes (Table 4). The most recent common ancestor node for *Poulsenia armata*, *Olmedia aspera*, and *Maquira costaricana* (in Moraceae) was associated with high manganese (Table 4). In addition, *Protium* (in Burseraceae) was associated with high manganese (Table 4). However, as summarized in the footnotes of Table 5, a number of groups showed association with more than one trait, as is the case for *Protium*, which was also significantly associated with low extractable phosphorus as mentioned above. A number of nodes also showed association with suites of traits as evaluated through the PCA axes (Table 5) and pH. The range of pH in this study is unlikely to directly influence plants but pH can be considered as an important proxy for nutrient and element availability. The node shared by Salicaceae and Clusiaceae was associated with low soil pH, as was the node uniting only Salicaceae and a number of nodes within Salicaceae (Table 3). Meliaceae, on the other hand, showed a significant association with high pH (Table 4).

## Discussion

The present study asked whether closely related tropical tree species tend to share similar soil associations in a tropical forest. Overall there was little to no phylogeny-wide evolutionary signal in soil nutrient/element associations, however the results from individual nodes within the phylogeny show that medians for a number of soil metrics had significant signal, and these instances may be important for understanding plant distributions at the local-scale in highly diverse tropical forest. This suggests that previous phylogenetic analyses of BCI assemblages that found significant phylogenetic structuring in different habitats [16], [19] may be partially explained upon the basis of phylogenetically conserved soil nutrient and element associations. However, because many soil variables within the dataset are correlated (Table 7 in [2]), and we could not account for species aggregation due to dispersal limitation, relationships between soil variables and taxa may not be causal. Below we discuss the results with respect to the association between individual soil variables and particular clades to highlight how plant-soil associations may drive the maintenance of diversity and the distributions of tropical genera and families.

### Aluminum

Aluminum is nonessential for plant growth and can be toxic, especially at pH less than 5.5 [41]. Lowland tropical forests supported by highly weathered soils often have high levels of extractable aluminum and relatively low soil pH, and floras of many forests on acidic soil have been suggested to confer tolerance to this element [41], [42]. The range of soil pH values, which are below 5.5, indicate that aluminum toxicity could be a concern in the BCI 50-ha plot soil. Moreover, the range of Mehlich-3 extractable aluminum was large (283–1563 mg kg^−1^, based on the 20m×20m scale data), suggesting that the potential of toxicity could vary.

This analysis detected an association between high extractable aluminum and the node shared by Myrtaceae and Vochysiaceae, *Eugenia* and *Chamguava* (in Myrtaceae), nodes within *Ficus* (in Moraceae) and a node shared by two species of *Psychotria* (Rubiaceae) (Table 4). To different degrees, all of these taxa with the exception of *Ficus* have been shown to be aluminum accumulators, which is one known mechanism for tolerating this potentially toxic element [43]. Vochysiaceae is well known to be a family characterized by ability to accumulate aluminum while species within Myrtaceae, and species within *Eugenia*, are noted as sometimes being aluminum accumulators [12]. Rubioideae, which includes *Psychotria*, is also noted as a group of aluminum accumulators [44], [45]. However, out of the 12 species of *Psychotria* present in the plot, only one node shared by several species of *Psychotria* was found to be significantly associated with high extractable aluminum and this node did not include *P. horizontalis*, which has over 3,000 individuals in the dataset.

It was interesting that Melastomataceae, which is a family well established as being aluminum accumulating [46], was not detected as having significant association with aluminum in this analysis. We note, however, that across the plot, the group could have a lower median of extractable aluminum yet still show preferential success in high aluminum areas compared to other taxa. In such a case an association would not be detected, although aluminum tolerance could still be an important strategy for taxa within this family. Although associations between Melastomataceae (and groups within this family) and aluminum were not detected in the current work, results do suggest that associations between aluminum and groups highlighted in this analysis, such as Myrtaceae and Vochysiaceae, *Eugenia* and *Chamguava*, and some groups of *Ficus* and *Psychotria*, could be important in determining local-scale plant distribution in BCI and other tropical forests that also show both high concentrations and large ranges of extractable aluminum.

### Manganese

Manganese is an essential plant nutrient but can be toxic or induce other deficiencies when present in high concentrations [47]. Comparing the extractable manganese levels associated with the nodes showing significant association with high manganese in this analysis (which include the node hosting *Poulsenia armata*, *Olmedia apsera and Maquira costariacana* (in Moraceae), the node shared by three *Zanthoxylum* species (in Rutaceae), and *Protium* (in Burseraceae) (Table 4)) shows that the high manganese levels in this study are near levels that have been considered toxic in a tropical agricultural system which also used a Mehlich-3 extract [48]. However, clearly the value of direct comparisons of extractable manganese among systems is limited because plant ability to tolerate high levels of manganese is known to differ by species [49], [50] and the effects of excess manganese can be dependent on concentrations of other ions [50]. Another line of evidence for the potential of excess manganese to influence plant distributions comes from temperate zones where high concentrations of manganese have been shown to negatively affect forest tree seedling performance [51], [52], [53], [54].

Out of the three forests investigated in John et al. [2], the BCI average of extractable manganese was more than twice that for Yasuni (a lowland forest in Ecuador), and nearly two magnitudes greater than that of La Planada (a montane forest in Columbia; Table 2 in [2]). Although John et al. [2] did not find manganese to have a relatively strong effect on a species analysis of niche structure compared to other nutrients/elements for BCI, the effect of manganese tended to be greater on BCI compared to the other two sites (Figure 3 in [2]). Because some tropical forest soils are characterized by high levels extractable manganese, and negative effects of excess manganese on plant performance have been shown to vary on at least a species level, the potential role of manganese in structuring lowland tropical forests that show large ranges in extractable manganese concentrations is worthy of future consideration.

### Phosphorus

Phosphorus is an important nutrient that is considered to have relatively low availability in highly weathered soils [55] and is thought to limit ecosystem processes in some tropical forests [13], [56], [57]. Although soil phosphorus is relatively high on BCI (for example, see total soil phosphorus data in [58]), our results suggest that if taxa such as Apocynaceae are sensitive to low phosphorus availability, phosphorus limitation may exist on a taxon specific level, even if a community as a whole is not limited. Analyses also detected that the large clade of Laurales and Magnoliales was associated with low extractable phosphorus, suggesting this clade may be relatively less sensitive to low levels of this nutrient, which could be due to lower requirements or more effective acquisition strategies.

Taxon specific access to different forms of phosphorus, which could be related to root fungal associations, may be one mechanism linking taxa with phosphorus availability [59]. The large majority of tropical trees associate with arbuscular mycorrhizae [60], which is a wide spread plant-fungal association that is thought to be ancestral [61] and can improve phosphorus acquisition [62], [63]. However, different arbuscular mycorrhizae have been shown to vary in their levels of benefit to different host species [64], [65] and plant species have been documented as having nonrandom associations with hosts [66], [67]. Although a great deal about the diversity of arbuscular fungi, the functional significance of this diversity and trends among host taxa remains unknown [68], investigating differences in arbuscular associations among groups, such as the Laurales and Magnoliales compared to Apocynaceae, could be especially interesting given the results of this study.

We do note, however, that the node shared by Laurales and Magnoliales was also significantly associated with slope (see below for discussion on slope) and the suite of traits influencing PCA axis 3, which includes low phosphorus and slope in addition to high aluminum and magnesium (Table 1). Similarly, *Protium*, another well represented group in this dataset (in Burseraceae), was also found to be significantly association with low extractable phosphorus, suggesting this group too may have strategies for dealing with lower phosphorus availability. However, *Protium* was also significantly associated with high manganese, as discussed in the previous section. These results highlight the fact that this study is not based on experimental manipulations and we cannot conclude if associations are the result of directly related mechanisms, random correlations, or interactions between a number of traits. Our results suggest that a trade-off between an ability to tolerate lower phosphorus levels and compete in more favorable conditions may exist within the BCI plot, but this remains to be experimentally tested.

### Slope

Mechanisms underpinning plant association with slope may be related to water availability and/or correlations with nutrients/elements. Sloped areas have been shown to be related to increased water potential during the dry season compared to plateau areas [32], [33], and species densities on drier plateaus compared to wetter slopes have been shown to be negatively correlated with drought sensitivity [69]. Within the BCI plot, slope is also known to have strong correlations with nutrients and elements (Table 7 in [2]). Sloped areas are well represented in the BCI plot with over 11 hectares having a slope greater than or equal to 7 degrees (Table 1 in [70]; slope range used in this study was 0.03 to 13.52 degrees, based on 20 m×20 m data). In comparison, areas categorized as plateau cover 31.6 ha of the plot, but note that 1.2 ha are categorized as swamp (also Table 1 in [70]), which could alter expectations for how water availability might be linked to a node-specific association with low slope in this study.

For the BCI community, association with slope has been well established at the species level [69], [70], [71], [72], [73], [74] and studies in many other systems have found species association with topography (for example, see [75], [76], [77] but also see [78]). The current analysis, which found that the node shared by Laurales and Magnoliales was significantly associated with higher slope, while Salicaceae was associated with low slope, suggests that patterns with slope could extend well beyond the species level to large clades that are well represented in most tropical forest communities.

### Tolerance and trait categories

Phylogenetic studies most commonly focus on traits that can be placed in one of five categories: life history, physiological, morphological, behavioral or ecological traits. Behavioral and ecological traits have, in general, been found to be more labile than other traits [22], [79]; however, tests for ecological traits, such as precipitation and mean annual temperature, are less common than those for behavioral traits. For example, Blomberg et al. [22] considered categories of life history, physiological, morphological and behavioral traits but ecological traits received little emphasis because of small sample sizes. In order to compare phylogenetic signal among traits and phylogenies, Blomberg et al. [22] developed a K statistic, which is a descriptive statistic that indicates the degree of trait similarity among closely related species relative to expectations from a Brownian motion model of trait evolution. Behavioral traits were found to be very labile, resulting in low K values [22], which suggests that expectations for the K statistic vary with trait category. Blomberg et al. [22] suggest that less similarity than expected from Brownian motion (K less than unity) could be the result of some but not all members of a group of species adapting to an environmental condition.

The current study focuses solely on soil characteristics, and we consider extractable aluminum, manganese, and phosphorus to be ecological traits, similar to mean annual precipitation and temperature as considered by others. We suggest that lower phylogeny-wide similarity than expected from Brownian motion would be expected in the case of evolution to ecological tolerance traits. However, for this category of traits, we propose that a low K statistic does not necessary imply that species within taxonomic groups have not evolved similarly with regard to the ecological trait of interest. Rather, similar to the reasoning underlying our hypothesis of lack of phylogeny-wide signal for soil variables, we suggest that phylogeny-wide statistics for ecological traits require a distinct interpretation. Because ecological traits are characterized by a disconnect between the metric measured and the mechanisms that would underlie response to the ‘trait’, trait values for some groups of species will necessarily be unconstrained and have large ranges. For example, consider a system where potentially toxic concentrations are limited to high end of the gradient. Species within a group displaying tolerance would have either 1) similar average soil trait values on the extreme end of the tolerance gradient, reflecting both their ability to tolerate such conditions and an associated trade-off related to performance under more favorable conditions or 2) a larger range of soil trait values which is biased toward but is not limited to values at the extreme end if a trade-off did not exist. In both cases, and especially the former case, the tolerant species would have averages that are more similar to the extreme than expected by chance. On the other hand, trait values for non-tolerant taxa would span a larger range of less extreme values. In turn, the phylogeny-wide K statistic would be less than unity yet the phylogeny could show node-specific associations in the trait value. If on the other hand, the measured trait were the mechanism conferring the tolerance (i.e., a physiological trait), the species found on the large range of less extreme soil values could show a constrained trait value and a higher K statistic could be expected.

We suggest this may be the case for the trait of extractable soil aluminum in the current study, as it had a K statistic of less than unity (K = 0.80 with marginal significance of p = 0.09; Table 2) and node-specific results from the AOT showed a number of significant nodes associated with high aluminum. Again, it is important to note that the details of the tolerance related trait metric are key in determining expectations for the category of ecological tolerance. Studies that use a ranking of tolerance as the trait metric [80], as opposed to using the value of the environmental variable to which the organism is responding, would not follow the same expectations. Similarly, studies investigating tolerance and focusing on physiological adaptations to environmental stress as the trait variables would fall in the category of physiological traits, altering expectations for trait lability and K statistics.

### Conclusion

The present work demonstrates that distributions of some plant taxa are associated with local-scale differences in soil variables when evaluated at individual nodes within the phylogenetic tree, but they are not detectable by phylogeny-wide signal. Some of the most intriguing results suggest aluminum and manganese, both potentially toxic elements, may structure plant distribution at the local-scale, as suggested by the significant associations between high extractable aluminum and the node shared by Vochysiaceae and Myrtaceae, in addition to the node shared by *Eugenia* and *Chamguava*, and associations between high manganese and *Poulsenia armata*, *Olmedia apsera* and *Maquira costariacana* (in Moraceae), the node shared by three *Zanthoxylum* species (in Rutaceae), and *Protium* (in Burseraceae). In addition, this study suggests slope may be an important variable underlying the distribution of Laurales and Magnoliales, a large clade of plants, and Salicaceae, a well represented family.

The BCI 50-ha plot site was chosen to limit abiotic variation [70], which suggests that similar analyses of local-scale plant-soil associations in plots that are randomly laid out could detect more local-scale associations than found here. The results presented in this study highlight associations that are most promising for future work aimed at understanding the role of edaphic factors and evolutionary history in determining community assemblages, and the maintenance of diversity, at the local-scale. The degree to which associations are mechanistically underpinned by the soil variables highlighted in this analysis, versus being the result of correlations between multiple edaphic variables and other environmental factors, remains to be determined. In addition, understanding the extent to which patterns uncovered in this analysis hold among tropical forests is a valuable research direction.

## Supporting Information

Figure S1 Extractable soil phosphorus. Mehlich-3 extractable soil phosphorus values (natural log transformed) are shown mapped on the molecular phylogeny for the BCI 50-ha plot.
(42.76 MB EPS)

Figure S2 Extractable soil aluminum. Mehlich-3 extractable soil aluminum values are shown mapped on the molecular phylogeny for the BCI 50-ha plot.
(10.72 MB EPS)

## References

[1] Sollins P (1998). Factors influencing species composition in tropical lowland rain forest: does soil matter?. Ecology.

[2] John R, Dalling J, Harms K, Yavitt JB, Stallard R (2007). Soil nutrients influence spatial distributions of tropical tree species.. Proc Natl Acad Sci USA.

[3] Gentry A (1988). Changes in plant community diversity and floristic composition on environmental and geographical gradients.. Ann Mo Bot Gard.

[4] Clark DB, Palmer MW, Clark DA (1999). Edaphic factors and the landscape-scale distributions of tropical rain forest trees.. Ecology.

[5] Tuomisto H, Ruokolainen K, Yli-Halla M (2003). Dispersal, environment, and floristic variation of western Amazonian forests.. Science.

[6] Tilman D (1988). Plant strategies and the dynamics and structure of plant communities.

[7] Fine PVA, Daly DC, Villa G, Mesones I, Cameron KM (2005). The contribution of edaphic heterogeneity to the evolution and diversity of Burseraceae trees in the western Amazon.. Evolution.

[8] Phillips OL, Vargas PN, Monteagudo AL, Cruz AP, Zans MEC (2003). Habitat association among Amazonian tree species: a landscape-scale approach.. J Ecol.

[9] Svenning JC, Kinner DA, Stallard RF, Engelbrecht BMJ, Wright SJ (2004). Ecological determinism in plant community structure across a tropical forest landscape.. Ecology.

[10] Davies SJ, Tan S, LaFrankie JV, Potts MD, Roubik D, Sakai S, Hamid Karim AA (2005). Soil-related floristic variation in the hyperdiverse dipterocarp forest in Lambir Hills, Sarawark.. Pollination Ecology and Rain Forest Diversity.

[11] Russo SE, Davies SJ, King DA, Tan S (2005). Soil-related performance variation and distributions of tree species in a Bornean rain forest.. J Ecol.

[12] Jansen S, Broadley MR, Robbrecht E, Smets E (2002). Aluminum hyperaccumulation in angiosperms: A review of its phylogenetic significance.. Bot Rev.

[13] Vitousek PM, Farrington H (1997). Nutrient limitation and soil development: Experimental test of a biogeochemical theory.. Biogeochemistry.

[14] Webb CO (2000). Exploring the phylogenetic structure of ecological communities: An example for rain forest trees.. Am Nat.

[15] Webb CO, Ackerly DD, McPeek MA, Donoghue MJ (2002). Phylogenies and community ecology.. Annu Rev of Ecol Syst.

[16] Kembel SW, Hubbell SP (2006). The phylogenetic structure of a neotropical forest tree community.. Ecology.

[17] Swenson NG, Enquist BJ, Pither J, Thompson J, Zimmerman JK (2006). The problem and promise of scale dependency in community phylogenetics.. Ecology.

[18] Swenson NG, Enquist BJ, Thompson J, Zimmerman JK (2007). The influence of spatial and size scale on phylogenetic relatedness in tropical forest communities.. Ecology.

[19] Kress WJ, Erickson DL, Jones FA, Swenson NG, Perez R (2009). Plant DNA barcodes and a community phylogeny of a tropical forest dynamics plot in Panama.. Proc Natl Acad Sci USA.

[20] Swenson NG, Enquist BJ (2009). Opposing assembly mechanisms in a Neotropical dry forest: implications for phylogenetic and functional community ecology.. Ecology.

[21] Condit R, Ashton PS, Baker P, Bunyavejchewin S, Gunatilleke S (2000). Spatial patterns in the distribution of tropical tree species.. Science.

[22] Blomberg S, Theodore Garland J, Ives A (2003). Testing for phylogenetic signal in comparative data: behavioral traits are more labile.. Evolution.

[23] Zimmerman JK, Wright SJ, Calderon O, Pagan MA, Paton S (2007). Flowering and fruiting phenologies of seasonal and aseasonal neotropical forests: the role of annual changes in irradiance.. J Trop Ecol.

[24] Baillie I, Elsenbeer H, Barthold F, Grimm R, Stallard R (2006). Semi-detailled soil survey of Barro Colorado Island, Panama.. http://biogeodbstrisiedu/bioinformatics/bci_soil_map/.

[25] Barthold F, Stallard R, Elsenbeer H (2008). Soil nutrient-landscape relationships in a lowland tropical rainforest in Panama.. Forest Ecol Manag.

[26] Condit R, Hubbell S, LaFranckie J, Sukumar R, Manokaran N (1996). Species-area and species-individual relationships for tropical trees: a comparison of three 50-ha plots.. J Ecol.

[27] Condit R, Ashton P, Manokaran N, LaFrankie J, Hubbell S (1999). Dynamics of the forest communities at Pasoh and Barro Colorado: comparing two 50-ha plots.. Philos T Roy Soc B.

[28] Perez R, Aguilar S, Condit R, Foster R, Hubbell S (2005). Metodologia empleada en los censos de la parcela de 50 hectareas de la Isla de Barro Colorado, Panamá.. Centro de Ciencias Forestales del Tropico (CTFS) y Instituto Smithsonian de Investigaciones Tropicales (STRI).

[29] Dalling J, John R, Harms K, Stallard R, Yavitt J (2009). Soil maps of Barro Colorado Island 50-ha plot.. http://ctfssiedu/datasets/bci/soilmaps/BCIsoil.html.

[30] Harms KE, Dalling JW, Yavitt JB, Stallard R (2004). Forest dynamics plot soil sampling protocols.. http://www.ctfs.si.edu/data///documents/SoilSmplProtocols1_Harms.pdf.

[31] Mehlich A (1984). Mehlich 3 soil test extractant: A modification of Mehlich 2 extractant.. Commun Soil Sci Plan.

[32] Becker P, Rabenold PE, Idol JR, Smith AP (1988). Water potential gradients for gaps and slopes in a Panamanian tropical moist forests dry season.. J Trop Ecol.

[33] Daws M, Mullins C, Burslem D, Paton S, Dalling J (2002). Topographic position affects the water regime in a semideciduous tropical forest in Panama.. Plant and Soil.

[34] Webb CO, Donoghue MJ (2005). Phylomatic: tree assembly for applied phylogenetics.. Mol Ecol Notes.

[35] Moles A, Ackerly D, Webb C, Tweddle J, Dickie J (2005). A brief history of seed size.. Science.

[36] Swenson N, Enquist B (2007). Ecological and evolutionary determinants of a key plant functional trait: wood density and its community-wide variation across latitude and elevation.. Am J Bot.

[37] Webb CO, Ackerly DD, Kembel SW (2008). Phylocom: software for the analysis of phylogenetic community structure and character evolution.. http://www.phylodiversity.net/phylocom/phylocom_manual.pdf.

[38] Blomberg SP, Garland T (2002). Tempo and mode in evolution: phylogenetic inertia, adaptation and comparative methods.. J Evolutionary Biol.

[39] Webb C, Ackerly D, Kembel S (2008). Phylocom: software for the analysis of phylogenetic community structure and trait evolution.. Bioinformatics.

[40] Kembel SW, Ackerly DD, Blomberg SP, Cornwell WK, Cowan PD Picante: R tools for integrating phylogenies and ecology.. Bioinformatics.

[41] Jansen S, Watanabe T, Dessein S, Robbrecht E, Smets E, Hemsley AR, Poole I (2004). The evolution of aluminum accumulation in angiosperms.. The Evolution of plant physiology.

[42] Webb L (1954). Aluminium accumulation in the Australian-New Guinea flora.. Aust J Bot.

[43] Ma JF, Ryan PR, Delhaize E (2001). Aluminium tolerance in plants and the complexing role of organic acids.. Trends Plant Sci.

[44] Jansen S, Dessein S, Piesschaert F, Robbrecht E, Smets E (2000). Aluminium accumulation in leaves of Rubiaceae: systematic and phylogenetic implications.. Annals of Botany.

[45] Stevens PF Angiosperm Phylogeny Website.. http://www.mobot.org/MOBOT/research/APweb/.

[46] Jansen S, Watanabe T, Smets E (2002). Aluminium accumulation in leaves of 127 species in Melastomataceae, with comments on the order Myrtales.. Ann Bot.

[47] Marschner H (1986). Mineral nutrition of higher plants.

[48] Hue NV, Vega S, Silva JA (2001). Manganese toxicity in a Hawaiian oxisol affected by soil pH and organic amendments.. Soil Sci Soc Am J.

[49] Morris H (1949). Minimum concentrations of manganese necessary for injury to various legumes in culture solutions.. Agron J.

[50] El-Jaoual T, Cox DA (1998). Manganese toxicity in plants.. J Plant Nutr.

[51] McQuattie CH, Schier GA (2000). Response of sugar maple (Acer saccharum) seedlings to manganese.. Can J For Res.

[52] Kitao M, Lei TT, Nakamura T, Koike T (2001). Manganese toxicity as indicated by visible foliar symptoms of Japanese white birch (Betula platyphylla var. japonica).. Environ Pollut.

[53] St Clair SB, Lynch JP (2005). Element accumulation patterns of deciduous and evergreen tree seedlings on acid soils: implications for sensitivity to manganese toxicity.. Tree Physiology.

[54] Ducic T, Leinemann L, Finkeldey R, Polle A (2006). Uptake and translocation of manganese in seedlings of two varieties of Douglas fir (Pseudotsuga menziesii var. viridis and glauca).. New Phytol.

[55] Walker TW, Syers JK (1976). Fate of phosphorus during pedogenesis.. Geoderma.

[56] Kaspari M, Garcia MN, Harms KE, Santana M, Wright SJ (2008). Multiple nutrients limit litterfall and decomposition in a tropical forest.. Ecol Lett.

[57] Lambers H, Raven JA, Shaver GR, Smith SE (2008). Plant nutrient-acquisition strategies change with soil age.. Trends Ecol Evol.

[58] Yavitt J, Wieder R (1988). Nitrogen, phosphorus, and sulfur properties of some forest soils on Barro-Colorado Island, Panama.. Biotropica.

[59] Turner BL (2008). Resource partitioning for soil phosphorus: a hypothesis.. J Ecol.

[60] Smith SE, Read DJ, Harley JL (1997). Mycorrhizal symbiosis.

[61] Fitter AH, Moyersoen B (1996). Evolutionary trends in root-microbe symbioses.. Philos T Roy Soc B.

[62] Sanders FE, Tinker PB (1971). Mechanism of absorption of phosphate from soil by endogone mycorrhizas.. Nature.

[63] Smith SE (1982). Inflow of phosphate into mycorrhizal and non-mycorrhizal plants of Trifolium-subterraneum at different levels of soil phosphate.. New Phytol.

[64] van der Heijden MGA, Klironomos JN, Ursic M, Moutoglis P, Streitwolf-Engel R (1998). Mycorrhizal fungal diversity determines plant biodiversity, ecosystem variability and productivity.. Nature.

[65] Kiers ET, Lovelock CE, Krueger EL, Herre EA (2000). Differential effects of tropical arbuscular mycorrhizal fungal inocula on root colonization and tree seedling growth: implications for tropical forest diversity.. Ecol Lett.

[66] Vandenkoornhuyse P, Ridgway KP, Watson IJ, Fitter AH, Young JPW (2003). Co-existing grass species have distinctive arbuscular mycorrhizal communities.. Mol Ecol.

[67] Husband R, Herre EA, Turner SL, Gallery R, Young JPW (2002). Molecular diversity of arbuscular mycorrhizal fungi and patterns of host association over time and space in a tropical forest.. Mol Ecol.

[68] van der Heijden MGA, Scheublin TR (2007). Functional traits in mycorrhizal ecology: their use for predicting the impact of arbuscular mycorrhizal fungal communities on plant growth and ecosystem functioning.. New Phytol.

[69] Engelbrecht BMJ, Comita LS, Condit R, Kursar TA, Tyree MT (2007). Drought sensitivity shapes species distribution patterns in tropical forests.. Nature.

[70] Harms KE, Condit R, Hubbell SP, Foster RB (2001). Habitat associations of trees and shrubs in a 50-ha neotropical forest plot.. J Ecol.

[71] Hubbell SP, Foster RB, Sutton SL, Whitmore TC, Chadwick C (1983). Diversity of canopy trees in a neotropical forest and implications for conservation.. British Ecological Society Special Publications Series, Vol 2, Tropical Rain Forest: Ecology and Management.

[72] Hubbell SP, Foster RB, Diamond J, Case TJ (1986). Biology, chance and history and the structure of tropical rain forest communities.. Community Ecology.

[73] Hubbell SP, Foster RB, Soule ME (1986). Commonness and rarity in a neotropical forest: implications for tropical tree conservation.. Conservation Biology: the Science of Scarcity and Diversity.

[74] Comita LS, Condit R, Hubbell SP (2007). Developmental changes in habitat associations of tropical trees.. J Ecol.

[75] Tateno R, Takeda H (2003). Forest structure and tree species distribution in relation to topography-mediated heterogeneity of soil nitrogen and light at the forest floor.. Ecol Res.

[76] Aiba S, Kitayama K, Takyu M (2004). Habitat associations with topography and canopy structure of tree species in a tropical montane forest on Mount Kinabalu, Borneo.. Plant Ecology.

[77] Gunatilleke CVS, Gunatilleke I, Esufali S, Harms KE, Ashton PMS (2006). Species-habitat associations in a Sri Lankan dipterocarp forest.. J Trop Ecol.

[78] Hall J, McKenna J, Ashton P, Gregoire T (2004). Habitat characterizations underestimate the role of edaphic factors controlling the distribution of Entandrophragma.. Ecology.

[79] Böhning-Gaese K, Oberrath R (1999). Phylogenetic effects on morphological, life-history, behavioural and ecological traits of birds.. Evol Ecol Res.

[80] Niinemets U, Valladares F (2006). Tolerance to shade, drought, and waterlogging of temperate Northern Hemisphere trees and shrubs.. Ecol Monogr.

